# Electrochemical creatinine detection for advanced point-of-care sensing devices: a review

**DOI:** 10.1039/d2ra04479j

**Published:** 2022-10-27

**Authors:** Carlos Luis Gonzalez-Gallardo, Noé Arjona, Lorena Álvarez-Contreras, Minerva Guerra-Balcázar

**Affiliations:** Facultad de Ingeniería, División de Investigación y Posgrado, Universidad Autónoma de Querétaro Querétaro C. P. 76010 Mexico minbalca@yahoo.com.mx; Centro de Investigación y Desarrollo Tecnológico en Electroquímica S. C. Sanfandila, Pedro Escobedo Querétaro C. P. 76703 Mexico; Centro de Investigación en Materiales Avanzados S. C. Complejo Industrial Chihuahua Chihuahua C. P. 31136 Mexico lorena.alvarez@cimav.edu.mx

## Abstract

Creatinine is an amino acid derived from creatine catabolism at different steps of the body's organs, and its detection is significant because levels out of normal values are linked to some diseases like kidney failure. Normal concentration levels of creatinine in blood are from 45 to 110 μM, while in urine, typical concentrations range between 3.3 to 27 mM, and in saliva from 8.8 and 26.5 μM. Nowadays, the creatinine detection is carried through different spectroscopic-colorimetric methods; however, the resulting values present errors due to high interferences, delayed analysis, and poor stability. Electrochemical sensors have been an alternative to creatinine detection, and the electrochemical methods have been adapted to detect in enzymatic and non-enzymatic sensors, the latter being more relevant in recent years. Nanomaterials have made creatinine sensors more stable, sensitive, and selective. This review presents recent advances in creatinine electrochemical sensors for advances in point-of-care (POC) sensing devices, comprising both a materials point of view and prototypes for advanced sensing. The effect of the metal, particle size, shape and other morphological and electronic characteristics of nanomaterials are discussed in terms of their impact on the effective detection of creatinine. In addition, the application of nanomaterials in POC devices is revised pointing to practical applications and looking for more straightforward and less expensive devices to manufacture.

## Abstract 2

Creatinine is an amino acid derived from creatine catabolism at different steps of the body's organs, and its detection is significant because levels out of normal values are linked to some diseases like kidney failure.

## Graphical abstract

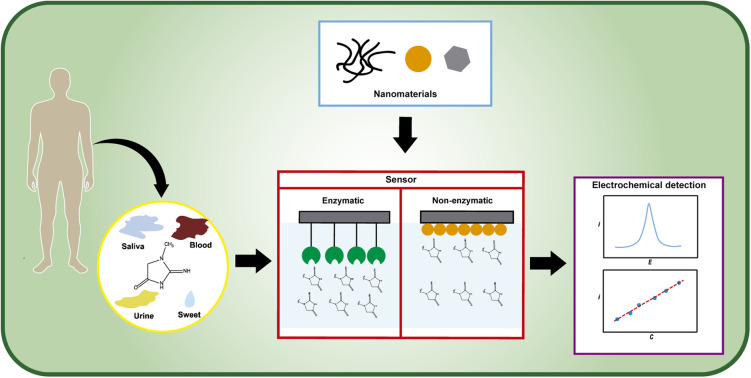

## 1. Introduction

**Fig. 1:**
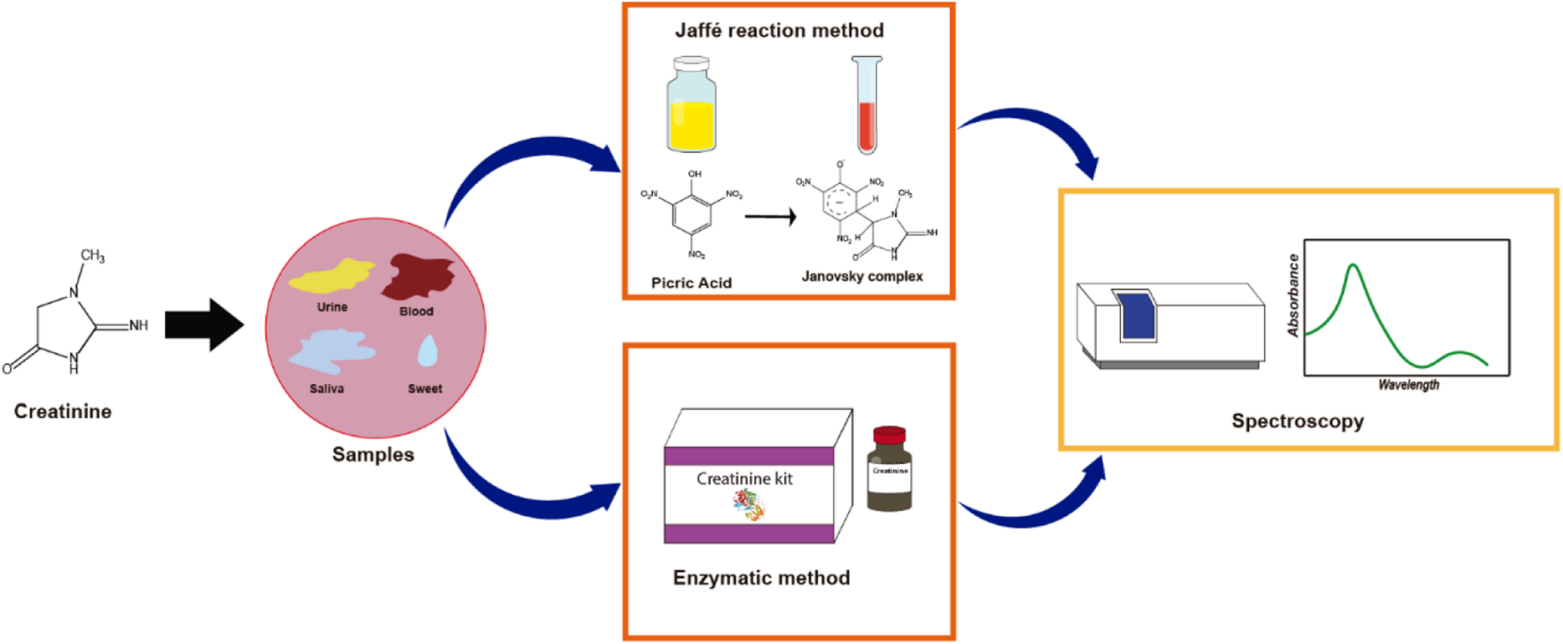
Creatinine detection method in clinical laboratories.

Nowadays, the increasing need for early diagnostic response and clinical monitoring with high sensitivity, selectivity, and fast throughput with sample analysis has been the main driver for the development of more efficient, low-cost, highly accurate, practical, and easy-to-use portable electrochemical (bio)sensors. Biosensors could be defined as a device based on specific biochemical reactions implicating isolated enzymes, immune systems, tissues, organelles, or whole cells during detecting electrical, thermal or optical signals of chemical compounds.^1^ Considering all these required innovations, it is clear that one of the challenges involves disruptive research and development, promoting the use of biomolecules as biomarkers of diseases from the point of view of not only diagnosis but especially disease prevention. Additionally, the detection of biomarkers like creatinine takes clinical relevance in the interpretation of the state of the body since it is related to muscle and kidney diseases. For example, kidney failure is a disease caused by the accumulation of waste in the body, including creatinine, resulting in an increase of its concentration.^2^ Creatinine (2-amino-1-methyl-5*H*-imidazole-4-one) is an amino acid compound derived from creatine catabolism, and because of this, the sensitive detection of creatinine is medically relevant. There are different strategies to detect it, the Jaffe reaction method being the most employed clinical analysis (Fig. 1). There are other typical methods like the spectrophotometric-colorimetric, which are subjected to many interferences, presenting low sensitivity,^3^ while enzymatic methods have higher selectivity but are relatively expensive.^4^

One of the main trends to detect creatinine is through electrochemical methods in enzymatic and non-enzymatic sensors, the enzymatic systems being the most reported electrochemical creatinine sensors because of their high selectivity. In the literature, there are electrochemical sensors for creatinine detection operating with one enzyme^5–7^ or three enzymes.^8–12^ However, it is worth mentioning that these systems present poor stability, sensitivity, and reproducibility, due to denaturalization of enzymes.^13^ In this regard, nanoparticles have helped face these disadvantages by increasing the surface area for immobilization or increasing sensitivity through a higher charge transfer.^14,15^ However, despite these improvements, enzymatic sensors are costly and tend to denature over time. The use of non-enzymatic sensors for creatinine detection has been proposed to overcome these drawbacks. The use of molecularly imprinted polymers is among the most studied non-enzymatic sensors.^16^ Actually, a nice review of nanomaterials for non-enzymatic detection of creatinine involving MIPs has been recently reported.^17^

Metallic nanomaterials have recently showed to be a good alternative to creatinine detection. Creatinine forms complexes with metals like silver, iron, and copper because the nitrogen of the aromatic ring of creatinine has a high affinity with the ions of these metals.^18^ With this premise, different research groups have conducted studies using Cu, Ag and Fe metallic nanoparticles to detect creatinine.^19,20^ Despite the advances in enzymatic and non-enzymatic sensors, the properties and advantages of nanomaterials have not yet been fully exploited. In this review, the electrochemical methods to detect creatinine are revised, followed by giving an approach of nanomaterials for creatinine detection involving a classical viewpoint (morphological aspects and electronic modifications through bimetallics and supports), and from a modern viewpoint but as a prospective. This is related to the limited literature dealing with the topic of defect/interface engineering on creatine detection. Then, the design, fabrication and operation of creatinine POC devices are revised and discussed.

## 2. Creatinine production during kidney failure

**Fig. 2:**
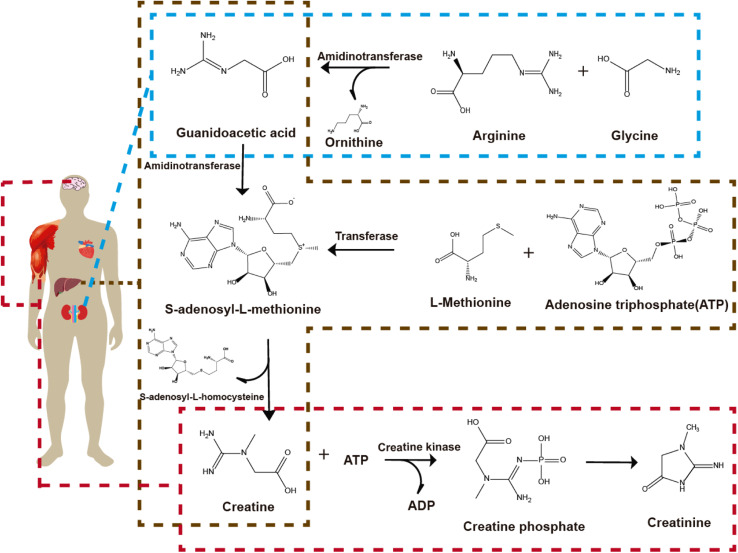
Schematic representation of creatinine metabolism in the human body.

The conversion of different compounds produced in the body is presented in Fig. 2. Arginine and glycine presented in the kidneys are converted to orthinine and guanidoacetic acid catalyzed by the enzyme amidinotransferase. Guanidoacetic acid is released from the kidneys and taken up by the liver. In the liver, l-methionine and adenosine triphosphate (ATP) are converted and catalyzed by the enzyme transferase to form *S*-adenosyl-l-methionine (SAMe). The enzyme methyl transferase converts guanidoacetic acid released from the kidneys and taken up by the liver with SAMe to *S*-adenosyl-l-homocysteine (SAH) and creatine. Finally, the creatine produced from the previous steps (and from the brain) and from ATP is catalyzed in the muscles with the enzyme creatine kinase to creatine phosphate and adenosine diphosphate (ADP) to form creatinine spontaneously.^21^ Creatinine is not only present in the blood but also in sweat, bile,^22^ urine, and saliva.^23^ When the kidneys fail, there is an increase of creatinine concentrations because the kidneys do not filter the blood effectively. Normal blood creatinine concentration levels are 45–90 μM in women and 60–110 μM in men,^24,25^ while a concentration up to 140 μM may indicate disease.^24^ Normal concentration levels of creatinine in urine ranged from 3.6 to 27 mM in men and 3.3 to 22.5 mM in women,^26^ while concentrations lower to 1.8 mM indicate anemia, hyperthyroidism, and kidney problems like kidney failure.^27^ Normal concentration levels of creatinine in saliva are from 8.8 to 26.5 μM, while concentrations of 16.7 μM up to 400 μM indicate kidney failure.^28,29^ In this regard, all non-enzymatic electrochemical sensors must meet these ranges.

## 3. Electrochemical techniques for detection and characterization

**Fig. 3:**
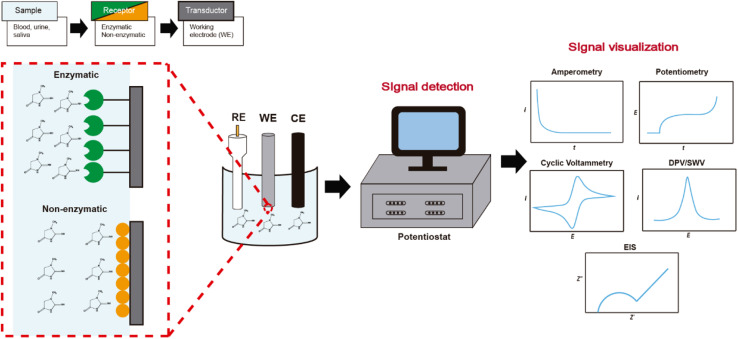
Schematic representation of electrochemical creatinine detection and signal visualization with different electrochemical techniques (amperometry, potentiometry, cyclic voltammetry, differential pulse voltammetry, square wave voltammetry, electrochemical impedance spectroscopy).

The detection and quantification of a biomarker follow the general scheme shown in Fig. 3. First, the biomolecule is immobilized in the electrochemical sensing process on the chemically modified electrode (receptor) based on electronic, semiconductor, or ionic conducting materials. Then, the response to the interaction is translated by the transducer, which finally sends the response in a suitable signal to identify and quantify. Electrochemical sensors have evolved according to different electrochemical detection techniques seeking greater sensitivity. In this manner, there are different reports sensing creatinine through amperometry, potentiometry, cyclic voltammetry, differential pulse voltammetry, and square wave voltammetry, each of them having particular features.

In the case of potentiometry for creatinine detection, it is worth to remember that this technique is based on measuring the potential difference between two electrodes: the reference electrode and the working electrode, in which no current flows between them, and because of this, any Faradaic reaction takes place. For creatinine, the most traditional specie detected by potentiometry is the ammonium ion product from the hydrolysis of creatinine by creatinine iminohydrolase (CIH),^5,6^ or creatinine deiminase (CD).^7,30^1
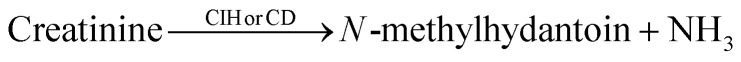


Huang *et al.* developed a multi-functional electrochemical detection chip (MFEDC), which can detect creatinine and urea through enzymatic reactions.^31^ For the detection of creatinine, the CD enzyme was used, which was mixed with aniline and *o*-phenylenediamine for physical entrapment, and the mixture was immobilized on the electrode by electropolymerization. The creatinine concentration range was 3160 to 39 000 μM, and the detection limit was 3160 μM. Pandey *et al.* developed a potentiometric creatinine sensor, where CI, CA, and urease enzymes were immobilized between layers of organically modified sol–gel glass and finally covered with electrochemically deposited *p*-toluene sulfonate doped polyaniline.^11^ The detection limit of the proposed sensor was 100 μM. Overall, the potentiometric sensors have drawbacks and limitations, such as interference from endogenous ammonia and other cationic substances,^11^ poor enzyme stability, and poor detection limits, such as 3160 μM,^31^ 20 μM,^32^ and 3 μM.^33^ In addition, temperature changes can affect detection, according to the Nernst equation and thus, a correct temperature analysis is required.

The amperometry technique consists of applying a potential difference between the working electrode and the reference electrode for a specific time. The oxidation or reduction reaction of the electroactive species gives a current response measured as a time function. Amperometry is one of the most used techniques in creatinine sensors due to its practicality, reaching limits of detection of 4.5 μM,^30^ 0.1 μM,^6^ 2.4 μM,^34^ and 0.01 μM^35^ in enzymatic sensors. In turn, it is also widely used in non-enzymatic sensors. Ciou *et al.* fabricated an electrode for creatinine detection with an ABTS–CNT|Nafion® composite.^36^ 2,2′-Azino-bis(3-ethylbenzothiazoline-6-sulphonic acid) (ABTS) and CNTs, which were mixed and then deposited on a screen-printed carbon electrode by drop casting to finally cover the electrode with a layer of Nafion®. The creatinine concentration range was from 0 to 20 000 μM, and the detection limit was found to be 11 μM. Other detection limits found in non-enzymatic sensors using amperometry were 0.22 μM^37^ and 0.083 μM.^38^

**Fig. 4:**
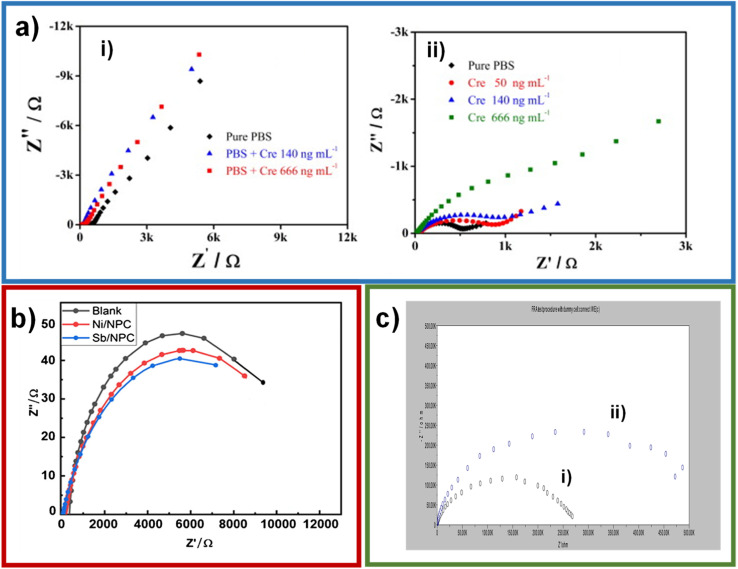
(a) Nyquist plots of EIS analysis of Cre–MA–MIP carbon paste electrode in phosphate buffer (pH 7.4) with the presence of creatinine (0, 50 140 and 666 ng mL^−1^) (i); Cre–MA–NIP carbon paste electrode in phosphate buffer (pH 7.4) with the presence of creatinine (0, 140 and 666 ng mL^−1^) (ii). Reproduced from ref. 39 with permission from Elsevier, Copyright (2013). (b) Impedance of Ni, Sb/NPC, and blank electrode. Reproduced from ref. 40 with permission from Elsevier, Copyright (2021). (c) Electrochemical impedance spectra (EIS) of bare GC electrode (i), CANPs/CINPs/SO_x_NPs modified GC electrode (ii). Reproduced from ref. 35 with permission from Elsevier, Copyright (2017).

Electrochemical impedance spectroscopy (EIS) is a complex resistance from the potential–current relationship, and valuable information about the system can be obtained through equivalent circuits such as the solution resistance, charge transfer resistance, and double layer capacitance. These components are plotted, obtaining the so-called Nyquist diagram. Reddy *et al.* employed impedance to corroborate the creatinine detection on molecularly imprinted polymer (MIP)-based non-enzymatic electrodes, comparing the results with a non-imprinted polymer electrode.^39^ The Nyquist diagrams showed that the non-imprinted polymer electrode did not present the semicircle of charge transfer resistance; the molecular imprinted polymer did with a value of 507 Ω. This behavior was attributed to creatinine molecules which are absorbed in the active sites of the polymeric matrix (Fig. 4a).

Jamil *et al.* performed an impedance analysis to observe the behavior of the Ni/NPC–GCE and Sb/NPC–GCE configurations on glassy carbon electrodes (GCE).^40^ The Sb/NPC–GCE had a lower charge-transfer resistance, which helped to improve the conductivity (Fig. 4b), which turns on an increase of the catalytic activity.^41–43^ Kumar *et al.* used impedance to compare charge-transfer resistances between GC electrode and CA NPs/CI NPs/Sox NPs modified GC electrode.^35^ It was observed that there is an increase in the resistance of the charge transfer in CA NPs/CI NPs/Sox NPs modified GC electrode in comparison with the naked GC electrode, indicating that the enzymes were immobilized in the sensor because the enzymes have poor electrical conductivity and hinder charge transfer (Fig. 4c). Also, to a lesser extent, impedance has been used in creatinine detections where resistance to charge transfer increases with the addition of creatinine.^44^

**Fig. 5:**
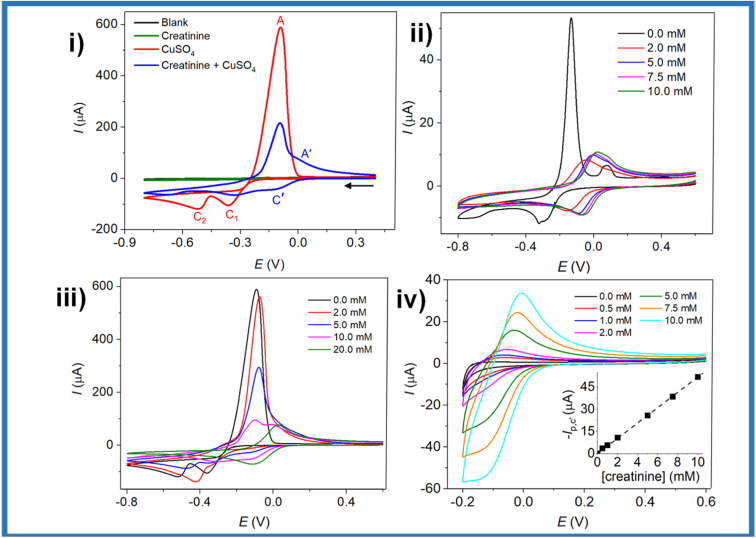
CVs of (black) blank 0.10 M K_2_SO_4_; (green) 7.5 mM creatinine; (red) 10.0 mM CuSO_4_; (blue) 10.0 mM CuSO_4_ and 7.5 mM creatinine at a glassy carbon electrode (i); CVs of varied creatinine concentrations (0–10 mM) in 1.0 mM CuSO_4_ and 0.10 M K_2_SO_4_ at a glassy carbon electrode (ii); CVs of varied concentrations of creatinine in the presence of 10.0 mM CuSO_4_ and 0.10 M K_2_SO_4_ at a glassy carbon electrode; potential window: −0.8 to 0.6 V (iii), and −0.2 to 0.6 V (iv); inset: a calibration plot of C′ peak currents *vs.* creatinine concentrations. Reproduced from ref. 45. This is an open access article distributed under the terms of the Creative Commons Attribution 4.0 License (CC BY, http://creativecommons.org/licenses/by/4.0/), which permits unrestricted reuse of the work in any medium.

The cyclic voltammetry is by excellence the most employed electrochemical technique for detection purposes because its simplicity, versatility and reproducibility. Ngamchuea *et al.* employed cyclic voltammetry to characterize the creatinine detection using CuSO_4_. For the system without creatinine, they observed a peak at −0.09 V (A) attributed to the oxidation of Cu^0^ to Cu^2+^ and two peaks at −0.37 V (C_1_) and −0.52 V (C_2_) belonging to the reduction of Cu^2+^ to Cu^+^ and from Cu^+^ to Cu^0^, respectively (Fig. 5i, red line).^45^ In the presence of creatinine, there is a reduction of the copper oxidation/reduction peaks and the appearance of two oxidation/reduction peaks at 0.02 V (A′) and −0.11 V (C′) (Fig. 5i, blue line). The C′ peak can be used for creatinine detection by narrowing the potential window, and the detection limit found by these authors was 35 μM, and the concentration range was 0–10 mM (Fig. 5ii–iv). It is worth mentioning that despite the advantages in characterization and detection of creatinine through cyclic voltammetry, the capacitive current coming from the accumulation of charges in the electrode–solution interface is a disadvantage, reducing its usefulness in detection by seeking lower limits of detection and concentration ranges. The limits of detection obtained by voltammetry have been 0.06 μM,^46^ 0.0746 μM,^19^ 0.002 μM,^43^ 6.5 μM^20^ and 0.3 μM,^47^ which are better than those obtained in potentiometry. However, within voltammetry, more sensitive techniques such as differential pulse voltammetry (DPV) or square wave voltammetry (SWV) can be chosen.

In the case of DPV, this technique allows to minimize the capacitive current, and, in this manner, concentrations of 10^−8^ M creatine can be measured.^48^ Wen *et al.* developed a molecular imprinted electrochemical sensor consisting of Fe_3_O_4_@polyanyline nanoparticles mixed in a solution with aniline and creatinine and attracted to a magnetic glassy carbon electrode. Then, the mixture attracted to the electrode is polymerized and treated with H_2_SO_4_ to remove creatinine and form the sensor.^49^ The DPV technique was used to detect creatinine using a molecular imprinting sensor consist of silver nanoparticles and reduced graphene oxide functionalized with polyoxometalate, obtaining a limit of detection of 0.0151 nM.^50^ Among the lowest limits of detection obtained by DPV are 76.3 nM,^51^ 0.61 mg dL^−1^ μM^−1^ ^52^ and 43 μM.^53^

In the case of SWV, likewise differential pulse voltammetry, the capacitive current is practically null, and thus, the advantages of selecting this as working electrochemical technique. However, there are not many works dealing with this technique. Viswanath *et al.* fabricated a sensor in which a mixture of Ag NPs and graphene oxide, which was electrochemically reduced on a glassy carbon electrode to form Ag NPs/rGO/GCE.^26^ The authors found that this material displayed a concentration range between 0.00001–0.00012 μM, and the detection limit was 7.43 × 10^−7^ μM. Overall, differential pulse voltammetry and square wave voltammetry have given relatively low concentration ranges and detection limits. This helps reduce the amount of sample needed and allow it to be subjected to pretreatments such as dilutions, which helps to adjust to the sensors' concentration ranges and reduce the influence of interferents.^54^ However, it is difficult to set these techniques in POC devices, and because of this, amperometry and cyclic voltammetry are still the most suitable techniques for prototypes of electrochemical creatinine sensors.

## 4. Nanomaterials in creatinine electrochemical sensors (CES)

Nanotechnology is a science that has been booming in recent years. Nanomaterials have specific properties different from bulk materials, making them attractive for their application in sensors. In a recently reported review,^17^ the incorporation of metallic nanoparticles to assemble complex transductors was discussed nicely. However, herein, we provide a different viewpoint attending the different properties given by the morphological and electronic modifications performed to metallic/non-metallic nanomaterials.

**Fig. 6:**
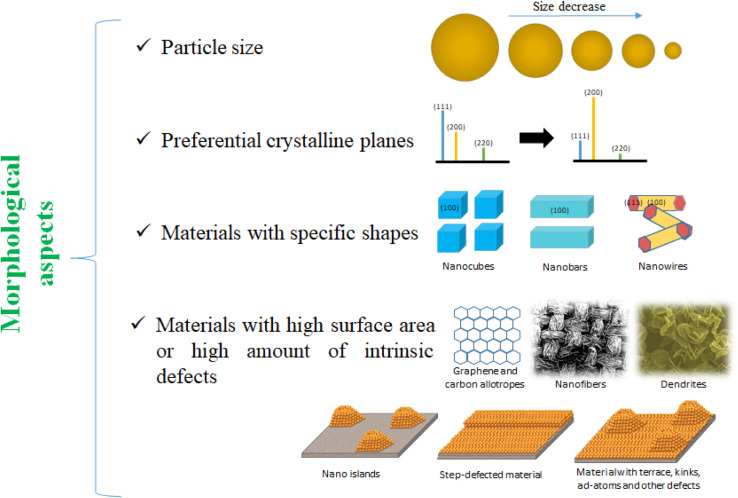
Morphological aspects of nanomaterials which can be modified/tuned to enhance the creatinine electrochemical detection.

From a classical viewpoint of nanomaterials, there are morphological (Fig. 6), and electronic characteristics, which can be modified. Morphological aspects like particle size, shape, and presence of preferential crystalline planes can modify the interaction with creatinine enhancing the detection limits. Likewise, the increment of surface area by using carbon allotropes as supports can boost the number of active sites available to interact with creatinine, while the enhancement of the amount of surface defects can promote changes in the electronic behavior of the nanomaterial.^55^ In a similar way, the electronic aspects of nanomaterials pursue to enhance the electron interaction to form creatinine-metallic center complexes. And, in this regard, modifications like using bimetallic nanoparticles, or enhancing the metal–support interaction can also allow a higher durability of POC devices.

In this section, the recent advances on nanomaterials for creatinine detection from this morphological/electronic viewpoint are summarized, while at the end of the section some perspectives are given from a modern materials science viewpoint involving surface/defect engineering.

### 4.1. Morphological aspects of nanomaterials for CES

Among the different morphological aspects of nanomaterial which can boost the detection of creatinine, the use of support materials with high surface area may be one of the most reported ways to improve the CES because the increase of the number of active sites. Within the wide range of support materials, multi-walled carbon nanotubes (MWCNTs) are one of the most interesting materials. MWCNTs have good mechanical properties, such as a higher tensile strength than steel.^56^ MWCNTs have excellent electrical properties, according to the hexagonal arrangement in the nanotube structure, which are higher than metals such as aluminum or copper.^57,58^ Lastly, MWCNTs can be modified by chemical, electrochemical, and plasma treatments.^59–61^ Using plasma and oxidants like nitric and sulfuric acids, MWCNTs can be functionalized, generating oxide groups, hydroxyl groups, carbonyl groups, and carboxyl groups.^57,62,63^ These characteristics of MWCNTs make them a versatile tool in their application to electrochemical sensors.

**Fig. 7:**
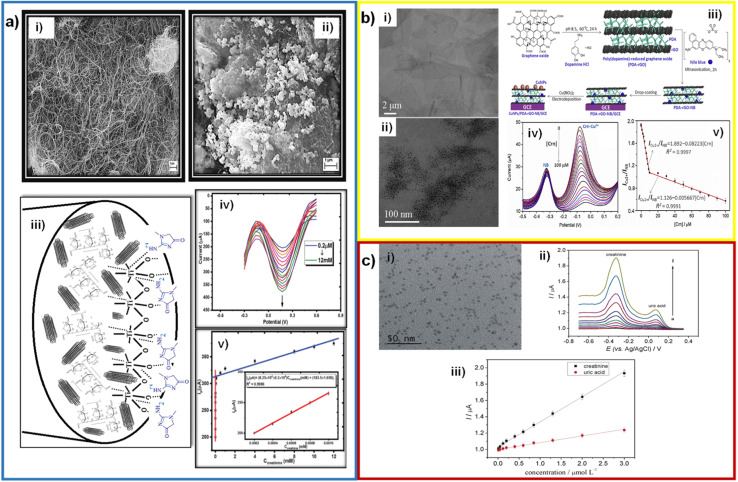
(a) SEM micrographs of MWCNTs before (i) and after (ii) modification with the Inu–TiO_2_ nanocomposite; mechanism of the interaction of creatinine with MWCNT–Inu–TiO_2_ sensor (iii); DPV curves of the sensor with creatinine additions and calibration plot (inset: first linear range) (v). Reproduced from ref. 41 with permission from Royal Society of Chemistry, Copyright (2018). (b) TEM micrographs of graphene oxide (i), PDA–rGO and Cu NPs/PDA–rGO–NB (ii); illustration of the fabrication procedures of an enzymeless ratiometric electrochemical sensor of creatinine based on Cu NPs/PDA–rGO–NB/GCE sensing platform (iii); SWV curves of the sensor with the addition of creatinine (iv) and the plotted linear relationships between *I*_Cu^2+^_/*I*_NB_ and [Crn] in the range of 0.01–100 μM (v). Reproduced from ref. 43 with permission from Elsevier, Copyright (2019). (c) TEM micrograph of GQDs nanocrystalline particles (i); SW voltammograms recorded at the new disposable electrochemical device for different creatinine and uric acid concentrations (ii) and calibration curves constructed for creatinine and uric acid at the PAD (iii). Reproduced from ref. 65 with permission from Elsevier, Copyright (2019).

Yazhini *et al.* used MWCNTs mixed with pectin to prepare the working electrode due to the high surface area and specific orientation, which provides more binding sites with pectin through OH bonds.^64^ Kalaivani *et al.* fabricated an electrode with a MWCNT/inulin–TiO_2_ composite, with limits of detection of 0.06 and 90 μM and concentration ranges of 0.2–1 and 50–12 000 μM.^41^ Inulin and TiO_2_ were mixed to form a biocomposite by Lewis's acid–base interactions. This biocomposite was used to modify MWCNTs, offering a high electroactive area and faster charge transfer (Fig. 7a). Fekry *et al.* fabricated an electrode composed of MWCNTs/Ag NPs/folic acid, with a limit of detection of 0.008 μM and a concentration range of 0.01–200 μM.^42^ These compounds were evenly mixed with graphite powder to form a carbon paste and used as an electrode. The detection mechanism they suggest is adsorption between the electrode containing a lone pair of electrons of folic acid or MWCNTs, and the lone pair of electrons of oxygen or nitrogen of creatinine, where direct oxidation of creatinine occurs.

Graphene is a material with a two-dimensional structure that occurs in the form of nanosheets. It has high hardness (similar to that of the diamond), elasticity, flexibility, density, and high thermal and electrical conductivity. Zhang *et al.* used rGO in a composite with MIP/Ag NPs/POM to increase the catalytic activity in the detection of creatinine, with limit of detection of 0.0000151 μM and concentration ranges of 0.00005–0.0015 μM.^50^ And, as before mentioned, part of this great LOD was attributed to the use of DPV as electroanalytical technique. Gao *et al.* deposited a Cu NPs/PDA–NB composite on reduced graphene oxide. PDA and NB were bound to the rGO surface by molecular interactions and π–π stacking (Fig. 7b).^43^ Anirudhan *et al.* fabricated a new molecularly imprinted polymer based on a TMSPMA–GO-*co*-HEMA/MMA composite, with a limit of detection of 16.6 μM and a concentration range of 44.2–265.21 μM.^52^ TMSPMA interacts with GO through its OH groups, forming the Si–O–C bond.

Quantum dots (QDs) are colloidal semiconductor particles with an approximate particle size of 1–20 nm. Having this size and being compared to the Bohr radius exciton, quantum dots acquire unique electrical and optical properties. Cincotto *et al.* modified carbon ink electrodes with graphene quantum dots and creatininase, with a limit of detection of 0.00375 μM and a concentration range of 0.010–3 μM.^65^ Additionally, quantum dots are characterized by the fact that electrons are confined in them in various directions.^66,67^ Hooshmand *et al.* modified a pencil graphite electrode with cadmium selenide quantum dots (CdSe QDs) by adsorbing them in the porous structure of graphite. This electrode has a limit of detection of 0.229 μM and a concentration range of 0.442–8840 μM.^68^ The CdSe QDs helped to have a high surface area and increase the catalytic activity of the electrode. The graphene quantum dots caused an increase in the electroactive area, helping to immobilize the enzyme creatininase with glutaraldehyde and improve the catalytic activity for the detection of creatinine (Fig. 7c). Ponnaiah *et al.* integrated carbon quantum dots into a WO_3_@GO nanocomposite, with a limit of detection of 0.0002 μM and a concentration range of 0.0002–0.112 μM.^69^ WO_3_ promotes charge transfer, and carbon quantum dots provide more active sites due to their high surface area.^70^

**Fig. 8:**
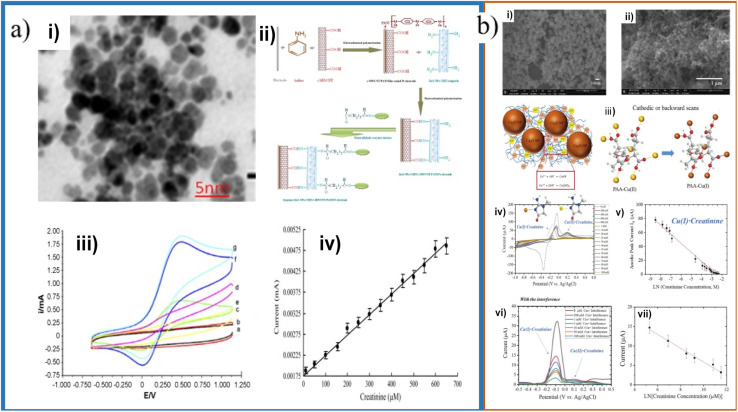
(a) TEM micrograph of ZnO NPs (i), schematic representation of chemical reaction involved in the fabrication of enzymes/ZnO NPs/CHIT/c-MWCNT/PANI/Pt electrode (ii); CVs of the electrode with different creatinine concentrations (iii) and calibration plot (iv). Adapted with permission.^71^ Copyright (2011), Elsevier. (b) SEM micrographs of synthesized bare Cu_2_O NPs (i) and PAA gel–Cu(ii)/Cu_2_O NPs/SPCE (ii); schematic representation of anodic stripping Cu^+^/Cu^2+^ of Cu_2_O NPs during forward (anodic) scans and the transformation of PAA–Cu(ii) to PAA–Cu(i) during cathodic scans (iii); CVs with different creatinine concentrations (iv); absolute anodic peak current responses *vs.* creatinine concentrations (v); DPV voltammograms obtained using PAA gel–Cu(ii)/Cu_2_O NPs/SPCE electrode for sensing different concentrations of creatinine with the interference of glycine, glucose, uric acid, ascorbic acid, and urea (vi) and calibration plot (vii). Adapted with permission.^20^ Copyright (2020), American Chemical Society.

Another important morphological aspect is the particle size. Nanoparticles are characterized by having an approximate size of 1 to 100 nm. Due to the nanometric size, nanoparticles exhibit a higher surface area/volume ratio than in bulk. As the surface area/volume ratio increases, the percentage of atoms on the surface and the surface forces become more dominant. The number of atoms on the surface increases as the particle size decrease, increasing the surface energy and their tendency to combine. This causes the surface atoms to be thermodynamically metastable or unstable. They tend to have their coordination numbers unsaturated. Another effect of size reduction is that the movement of electrons is hampered, called quantum confinement. This quantum confinement produces changes in electronic, optical, magnetic, and electromagnetic properties compared to bulk materials. Yadav *et al.* developed an electrode using ZnO nanoparticles in a PANI/c-MWCNTs/chitosan composite, with an electrode limit of detection and concentration range of 0.5 μM and 10–650 μM, respectively.^71^ ZnO NPs varied in size from 10 to 30 nm, and they were dispersed in chitosan and electrodeposited in the PANI/c-MWCNTs composite in order to increase charge transfer and help improve the electrode response and increase the sensitivity (Fig. 8a).

Fe_3_O_4_ NPs have unique properties such as strong superparamagnetic behavior and high biocompatibility that favor better delivery and recovery of biomolecules.^72,73^ These Fe_3_O_4_ nanoparticles with a size of 20 nm were dispersed in chitosan and electrodeposited with aniline, promoting a high charge transfer and a permeable surface.

Kasap *et al.* fabricated an electrode with gold nanoparticles/modified zeolite/creatinine deiminase, with a limit of detection of 5 μM and a concentration range of up to 2 mM. The Au NPs, of a size of 10 nm, increase the surface area of the electrode for the interaction of the NPs with the amino groups of the enzyme.^74^ Braiek *et al.* used gold nanoparticles in a polyvinyl alcohol/polyethyleneimine composite, with a limit of detection and concentration range of 2 μM and 10–600 μM, respectively. The gold nanoparticles were 23 nm in size and they allowed to increase the sensor's sensitivity in the conductometric hydrolase detection.^75^ Kalasin *et al.* developed an electrode with Cu_2_O NPs with a particle size of 98.4 nm in a polyacrylic acid gel–Cu^2+^ composite, with a limit of detection of 6.5 μM and a concentration range of 200–100 000 μM, and Cu_2_O NPs with a size of 49.2 nm in a Nafion®/polyacrylic acid gel–Cu^2+^ composite, with a limit of detection of 0.3 μM and a concentration range of 1–2000 μM (Fig. 10c).^20,47^ Gao *et al.* and Kalasin *et al.* used copper to detect creatinine because, as with silver and iron, creatinine has a high affinity for complexation with copper by the creatinine nitrogen.^18,76^ As can be observed from the previous work, small sized nanoparticles have been employed for CES displaying an enhancement in sensitivity, lowering the detection limits because the surface area enhancement, while nanomaterials like gold have being used to decrease the enzyme denaturation. However, there are no systematic works devoted to analyze the particle size effects on CES.

The particle shape arises from the growth of atoms on certain domains, and thus, predominant crystalline planes with different surface energies are common on these nanomaterials. It is well-know that this different in surface energies can promote changes on the adsorption and complex formation of different species of interest in electrochemical sensing. The use of nanoparticles with certain shapes has been limited on the ECS. Among the different literature, it is worth mentioning that most of the works reports the use of spherical shapes,^14,35,37,42,51,74^ hexagonal shapes,^14^ and sheet shapes^26,77^ to a greater extent.

**Fig. 9:**
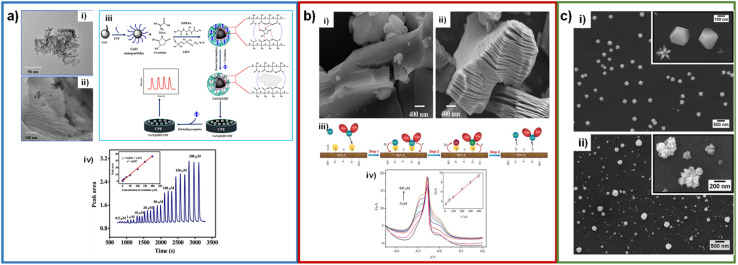
(a) TEM micrograph CuO (i) and CuO@MIP (ii); synthesis of CuO@MIP and CuO@MIP/CPE preparation procedure (iii); FIA gram of creatinine detection (inset: calibration plot) (iv). Adapted with permission.^38^ Copyright (2019), Elsevier. (b) SEM micrographs of Ti_3_AlC_2_ (i) and Ti_3_C_2_T_*x*_ (ii); schematic illustration of creatinine determination (iii); SWV responses of the microfluidic chip toward different concentrations of creatinine (inset calibration plot) (iv). Adapted with permission.^77^ Copyright (2018), John Wiley and Sons. (c) SEM micrographs of copper deposited on glassy carbon plates after electrodeposition at −0.8 V in 10.0 mM CuSO_4_ (i), 7.5 mM creatinine and 10.0 mM CuSO_4_ (ii). Adapted with permission.^45^ This is an open access article distributed under the terms of the Creative Commons Attribution 4.0 License (CC BY, http://creativecommons.org/licenses/by/4.0/), which permits unrestricted reuse of the work in any medium.

Nontawong *et al.* fabricated a creatinine sensor based on CuO NPs coated with a molecular imprinted polymer and deposited on a GC electrode, with a limit of detection of 0.083 μM and a concentration range of 0.5–200 μM. Creatinine and MAA functional monomer were mixed, then CuO NPs (spherical shape with a diameter of 11.3 ± 0.1 nm), DHEBA cross-linker, and AIBN initiator were added, forming a gel that was then polymerized, and the template (creatinine) removed to form CuO@MIP NPs. The CuO@MIP NPs had a highly rough and porous surface, having a larger surface area to absorb creatinine, aiding detection (Fig. 9a). In the presence of creatinine CuO@MIP/CPE presents an oxidation peak at 0.31 V, where it was proposed that oxidation of creatinine produces one electron and one proton per molecule of creatinine.^38^ Liu *et al.* developed a screen-printed electrode modified with MXene, with a limit of detection of 1.2 μM and a concentration range of 10–400 μM.^77^ This MXene has the form of nanosheets, which gives them a multilayer structure that helps increase the surface area and therefore helps increase catalytic activity and adsorb other elements that are needed for recognition as enzymes (Fig. 9b). Ngamchuea *et al.* studied the structure of copper–creatinine complex formation by SEM. In SEM micrographs, the copper deposit was studied at a stop potential of −0.8 V, where in the absence of creatinine, the copper deposit had an icosahedral shape. In contrast, with the presence of creatinine, the deposit had a cauliflower shape, similar to additives that are absorbed into copper, confirming the formation of copper–creatinine complexes (Fig. 9c). Nonetheless, the authors did not comment on the positive effect of the Cu shape.^45^

### 4.2. Electronic aspects of nanomaterials

**Fig. 10:**
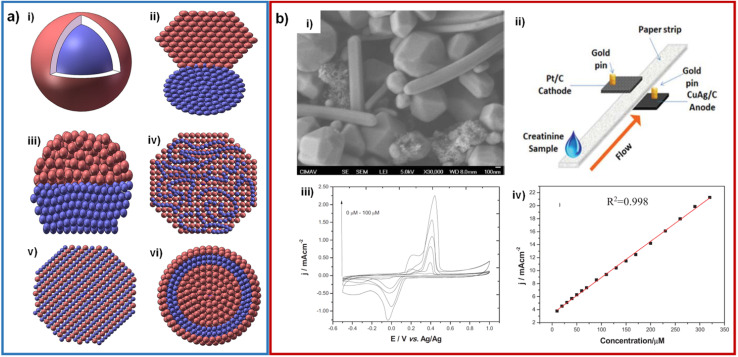
(a) Schematic representation of structures of bimetallic nanoparticles: core–shell (i), subclusters with small bond (ii), subclusters segregated (iii), mixed allay (iv), random alloy (v) and two shells core–shell NPs (vi). Adapted with permission.^79^ Copyright (2021), Taylor & Francis. (b) SEM micrograph of CuAg wire-shaped structures (i); design of the creatinine paper-based microfluidic fuel cell (ii); cyclic voltammetry of CuAg/CP at several creatinine concentrations, phosphate buffer pH = 7.4 at 50 mV s^−1^ (iii) and calibration curve for the analysis of creatinine (iv). Adapted with permission.^81^ Copyright (2019), IEEE.

As above mentioned, the electronic aspects of nanomaterials be able to promote a faster charge transfer, allowing a better creatinine electrochemical detection. Among the different strategies to enhance the electron transfer, the most reported has been through employing supports with high surface area (and high conductivity). However, bimetallic nanoparticles can assist on that purpose. Bimetallic nanoparticles can be formed with different morphologies and arrangements, such as core–shell, clusters or alloy (Fig. 10a).^78–80^

Until our knowledge, the only work related to bimetallics for CES was reported by our group. In such work a creatinine sensor was developed by synthesizing bimetallic CuAg nanoparticles (polygonal shape with a size of 500 μM and nanowires with a diameter of 200 nm) that were then mixed with Nafion®, carbon black, and isopropanol, forming a catalytic ink that was deposited on a carbon paper electrode.^81^ These CuAg bimetallic nanoparticles helped the metallic copper not to undergo rapid oxidation under ambient conditions. The concentration range obtained was 0–320 μM. In addition, these bimetallic nanoparticles were used in a fuel cell that generated a power density of 1 μW cm^−2^ (Fig. 10b).

Tables 1 and 2 were constructed with the aim of summarizing the findings before mentioned from a materials science viewpoint. In Tables 1 and 2 the characteristics of enzymatic and non-enzymatic sensors investigated in recent years are displayed. It is possible to observe from these tables that the size and the support material are the most investigated aspects of nanomaterials. This is because the size of the nanoparticles and the support materials promote a better kinetics and charge transfer, improving the sensor's sensitivity. The size effect has also been directly applied to the formation of enzymatic nanoparticles, improving their qualities compared to conventional enzymes. Support materials have also served as enzyme immobilization surfaces, giving stability to enzymatic sensors. The shape of nanomaterials and their crystallographic planes have been explored very little, being limited to the use of spherical nanoparticles. Finally, bimetallic nanoparticles have been addressed in only one investigation, so the catalytic improvements to their monometallic counterparts have not been taken advantage of. Therefore, it is essential that the application of nanomaterials extends to these last characteristics for the study of creatinine detection and to improve the properties of the sensors.

**Table 1:** Electrochemical creatinine detection on enzymatic-based nanomaterials^a^

Electrochemical technique	Transducer	Enzymes	Nanomaterial	Linear range (μM)	LOD (μM)	Response time	Samples	Ref.
Amperometry	c-MWCNT/PANI/Pt	CA, CI, SO	c-MWCNT	10–750	0.1	5 s	Human serum	13
Amperometry	Fe_3_O_4_ NPs/CHIT-*g*-PANI/Pt	CA, CI, SO	Fe_3_O_4_ NPs	1–800	1	2 s	Human serum	14
Amperometry	HRP/Fc/Au NPs/MWCNTs/Teflon	CA, CI, SO	Au NPs, MWCNTs	3–1000	0.1	19 s	Human serum	15
Potentiometry	PANI/*o*-PD/Pt	CD	—	3160–39 000	3160	20 min	Fetal bovine serum	31
Potentiometry	Nafion®/PVA–SbQ/pH-FET	CD	—	20–2000	20	2–3 min	Human serum	32
Potentiometry	PVC–NH_2_/graphite	Creatininase	—	80–100 000	3	10 s	Human serum	33
Amperometry	Ferrocenemethanol–SPCE	CA, CI, SO, HRP	—	5–1000	2.4	10 s	Human blood	34
Amperometry	ENPs/GCE	CA, CI, SO	ENPs	0.01–12	0.01	2 s	Human serum	35
Amperometry	PANI/Nafion®/Cu/SPE	CDI	Cu NPs	1–100	0.5	15 s	Human serum	54
Square wave voltammetry	GQDs/hexaammine–ruthenium(iii) chloride	Creatininase	GQDs	0.010–3	0.00375	—	Human urine	65
Amperometry	ZnO NPs/CHIT/c-MWCNT/PANI/Pt	CA, CI, SO	c-MWCNT, ZnO NPs	10–650	0.5	10 s	Human blood serum	71
Conductimetry	Au NPs/PV/PEI/Au	CD	Au NPs	2–600	2	3 min	Artificial blood serum	75
Cyclic voltammetry	CoCl_2_/SPCE	CDI	—	17.68–353.61	—	3 min	Human serum albumin	101
Amperometry	Nitrocellulose membrane, Pt	CA, CI, SO	—	0–10 000	—	6.9 min	Bovine serum albumin	107

a Abbreviations: HRP: horseradish peroxidase; MWCNT: multi-walled carbon nanotube; PANI: polyaniline; *o*-PD: *o*-phenylenediamine; CHIT: chitosan; PVC: poly(vinylchloride); Fc: ferrocene; SPCE: screen printed carbon electrode; PVA–SbQ: poly(vinyl alcohol), *N*-methyl-4(4′-formylstyryl)pyridinium methosulfate acetal; ENP: enzyme nanoparticles; GCE: glassy carbon electrode; DB30C10: dibenzo-30-crown-10; RH: rice husk; GQDs: graphene quantum dots.

**Table 2:** Electrochemical creatinine detection on non-enzymatic systems based on nanomaterials^a^

Electrochemical technique	Transducer	Nanomaterial	Linear range (μM)	LOD (μM)	Response time (s)	Samples	Ref.
CV	Copper/SPCE	—	6–378	0.0746	20 s	Human serum	19
CV/DPV	PAA gel–Cu(ii)/Cu_2_O NPs/SPCE	Cu_2_O NPs	200–100 000	6.5	—	Urine	20
SWV	rGO/Ag NPs/GCE	Ag NPs, rGO	0.00001–0.00012	7.43 × 10^−7^	—	Urine	26
Amperometry	CNT–ABTS/Nafion®/SPCE	CNTs	0–21 300	11	50 s	Urine	36
Amperometry	CuO/IL/ERGO/SPCE	CuO nanocrystals	10–2000	0.22	5 s	Human serum	37
Amperometry	CuO@MIP/CPE	CuO NPs	0.5–200	0.083	—	Urine	38
EIS	MIP/carbon paste electrode	—	0.18–5.92	0.18	—	Human serum and artificial urine	39
CV	Sb/NPC–GCE	Sb NPs, NPC	0.6–1.0	0.74	—	Human serum	40
DPV	CP/MWCNT–Inu–TiO_2_	TiO_2_ NPs, MWCNTs	0.2–1	0.06	—	Human serum	41
			50–12 000	90	20 s	Urine	
DPV	Ag NPs/MWCNTs/FA/CPE	Ag NPs, MWCNTs	0.01–200	0.008	1.5 s	Human serum and urine	42
CV	Cu NPs/PDA–rGO–NB/GCE	Cu NPs, rGO	0.01–100	0.002	—	Human serum and urine	43
DPV/EIS	MIP/Au–SCPE	—	0.00088–0.00884	0.00014	—	Urine	44
CV	Phosphotungstic acid/poly(ethyleneimine)/ITO	—	0.125–62.5	0.06	20 s	Urine	46
CV/DPV	Nafion®/polyacrylic gel–Cu^2+^/Cu_2_O NPs/SPCE	Cu_2_O NPs	1–2000	0.3	—	Saliva	47
DPV	Fe_3_O_4_@PANI/creatinine/aniline/MGCE	Fe_3_O_4_@PANINPs	0.02–1	0.00035	—	Human plasma and urine	49
DPV	MIP/Ag NPs/POM/rGO coated GCE	Ag NPs, rGO	0.00005–0.0015	0.0000151	—	Human serum, saliva	50
DPV	MIP/Ni@PANI NPs/MCGE	Ni@PANI NPs	0.004–0.8	0.0002	—	Urine	51
DPV	TMSPMA–GO-*co*-HEMA/MMA	GO	44.2–265.21	16.6	2 min	Human serum and urine	52
DPV	Fe^3+^/CB NPs/SPCE	CB NPs	100–6500	43	—	Urine	53
DPV	Pectin–MWCNT/CPE	MWCNTs	0.016–3.3	0.6241	—	Urine	64
DPV	CdS quantum dots/PGE	CdS quantum dots	0.442–8840	0.229	—	Human serum and urine	68
DPV	CDs/WO_3_@GO/GCE	CDs	0.0002–0.112	0.0002	—	Human blood and urine	69
SWV	MXene/Cu ions/SPCE	2D MXene Ti_3_C_2_T_*x*_ nanosheet	10–400	1.2	15 min	Human blood	77
DPV	PMB/PVAc/Cu/CNF/ACF	Carbon nanofibers CNFs	0.04–7.96	0.02	—	Human blood serum, saliva	78
CV	CuAg NPs/Nafion®/CB/CP	CuAg NPs	0–320	—	—	—	81
Amperometry	Polypyrrole/creatinine/Au	—	0–1000	40	5 min	Blood	99
CV/DPV	Fe–Cu–rGO@Ag	Fe–Cu–rGO nanocomposite	0.01–1000	0.01		Human blood	102

a Abbreviations: CV: cyclic voltammetry; DPV: differential pulse voltammetry; SWV: square wave voltammetry; EIS: electrochemical impedance spectroscopy; MIP: molecular imprinted polymer; PMB: poly methylene blue; PVAc: polyvinyl acetate; CNF: carbon nanofiber; ACF: active carbon fiber; POM: polyoxometalates; PDA: polydopamine; NB: Nile blue; TMSPMA: trimethyl silane propyl methacrylate; HEMA: 2-hydroxymethacrylate; MMA: methyl methacrylate; IL: ionic liquid; ERGO: electrochemically reduced graphene oxide; CP/CPE: carbon paste/carbon paste electrode; Inu: inulin; CB: carbon black; ABTS: 2,2′-azino-bis(3-ethylbenzothiazoline-6-sulphonic acid); FA: folic acid; PAA: polyacrylic acid; CDs: carbon dots; Sb/NPC: N-doped porous carbon antimony nanoparticle.

### 4.3. Perspectives from a modern materials science viewpoint

**Fig. 11:**
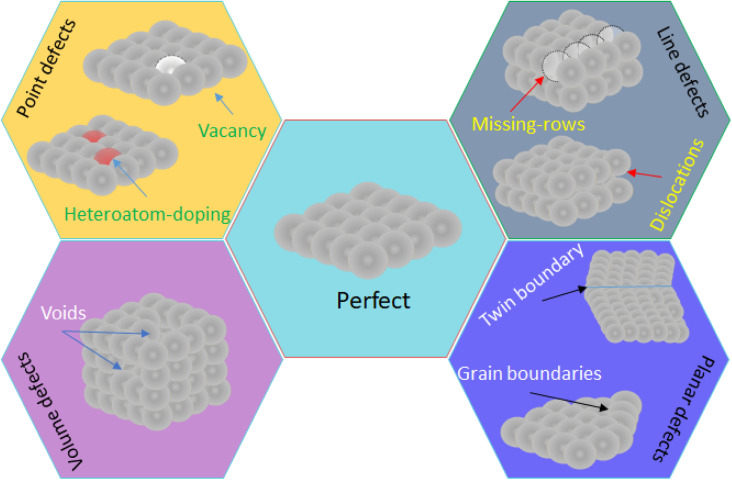
Illustration of the main surface defects in nanomaterials.

In the material science, there are some new findings pursuing to increase the number and reactivity of active sites. These fields of research are named as defect engineering and interface engineering, respectively. Defect engineering consist of controlling the surface defects (crystallographic, structural, topological, *etc.*) to change key parameters like the electronic behavior of nanomaterials, the coordination number of surface atoms, and the number of active sites. Defect engineering involves not only the promotion of point, line, planar and volume defects (Fig. 11),^82^ but also doping nanomaterials with heteroatoms, which can be metallic and non-metallic like sulfur, nitrogen, phosphorus, boron.^83^ Thus, the number of active sites enhances the velocity of the electrochemical reaction, while the intrinsic activity of active sites is achieved by doping with metallic/non-metallic heteroatoms because the electronic structure is modified, and by oxygen vacancies.^84^ In the case of the interface engineering, for instance, combines the defect engineering applied to the metal nanoparticle and the support. There are many works dealing with defect engineering on nanomaterials for different applications like energy conversion,^85^ energy storage,^86^ wastewater treatment,^87^ and electrochemical sensors.^88^ However, until our knowledge there are no works devoted to the study of defect/interface engineering on the electrochemical detection of creatinine.

## 5. Applications of creatinine sensors in point-of-care devices

The developments in biomarker sensors have the objective of being able to expand the rapid, economic and reliable diagnosis capacity that allows having devices practically in the doctor office or at home. An example of this type of development is the devices to measure the glucose level that can now be obtained very easily. In the case of creatinine detection, the most widely used procedure is the Jaffe procedure, which was designed as a result of the work of Max Jaffe in 1886,^89^ however this procedure is not specific for creatinine and uses acid picric, which is more explosive than TNT. Although the measurement of creatinine by this method is effective, simple and inexpensive, it is an outdated method and unsuitable for the requirements of modern clinical analysis. Since the 1950s, researchers have tried to design a new procedure for the analysis of creatinine in urine.^90^ Currently, desirable technologies must be point-of-care (POC), portable and inexpensive, which is motivated by the need for rapid diagnoses that can prevent complications in the patient health and the need to reduce the economy of care medical. Detection in POC systems must be a rapid method of detection without the need for samples to travel and be altered, or the need for trained analytical personnel to use complicated and/or expensive equipment. Depending on the nature of the sensing element, electrochemical sensors are generally classified in enzymatic sensors, enzyme-free and paper-based sensors.^91^ While there are many different methods for urinary creatinine analysis (GC-MS,^91^ capillary zone electrophoresis,^92^ LC-IDMS,^93^ diffuse reflectance UV/vis spectrometry,^94^*etc.*), few have evolved towards portability for POC diagnostics. There are currently POC creatinine devices on the market,^95^ some are multisensors that electrochemically detect a variety of target analytes, including creatinine, glucose, K^+^, Na^+^, lactose, Cl^−^ and Ca^2+^ with response times of 150 s; however, the sample size is 110 μL of blood for analysis, which is a high volume compared to that required for glucose sensors, therefore so this is a limiting factor. The available technology has an error of approximately 11.4% (ref. 95) and the detection level of creatinine in blood is 40 μM, which is well below the lower limit of normal for an adult male. Work has also been reported for the detection of serum creatinine using miniaturized capillary zone electrophoresis microfluidic devices with an integrated electrode and temperature indicator.^96^ Such a device is portable and therefore can be used as a POC device. In these systems, the patient's serum is used, so it cannot be easily used at home. Additionally, a drawback of this device is that the quantification limit is 300 μM.^96^ A typical healthy adult male has approximately 80 μM serum creatinine, and although the detection limit is approximately 100 μM with this device, it is not low enough for clinical use. This device is limited for use in patients with a very high level of creatinine in the blood. De Araújo *et al.*, used the Jaffe reaction electrochemically in an indirect way, quantifying the picrate anion, which is consumed when reacting with creatinine.^97^ This method was improved using screen-printed carbon electrodes detecting at healthy and unhealthy patient levels.^98^

**Fig. 12:**
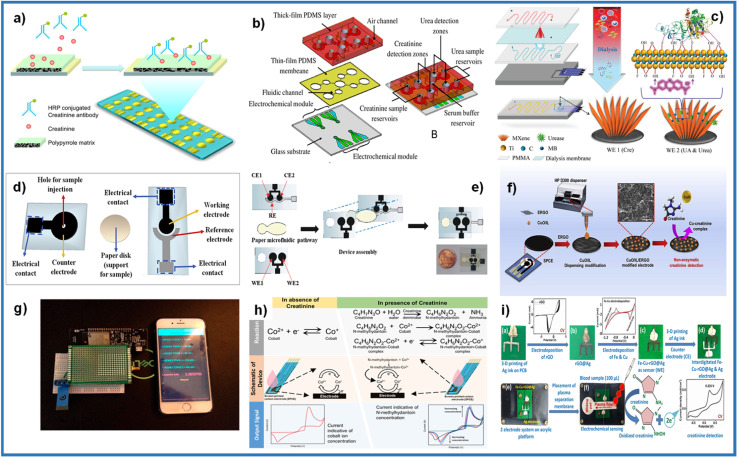
(a) Conducting polymer electrochemical sensor. Adapted with permission.^99^ Copyright (2012), American Chemical Society. (b) An exploded view of multi-functional electrochemical detection chip (MFEDC). Adapted with permission.^31^ Copyright (2011), John Wiley and Sons. (c) Fabrication of MXene-enabled microfluidic chip. Adapted with permission.^77^ Copyright (2018), John Wiley and Sons. (d) Separated components of electrochemical paper-based analytical device (ePAD). Adapted with permission.^53^ Copyright (2020), Elsevier. (e) Steps involving the construction of ePAD. Adapted with permission.^65^ Copyright (2019), Elsevier. (f) Paper-based analytical device (PAD) fabrication, modifying the working electrode with CuO/IL/ERGO for creatinine detection. Adapted with permission.^37^ Copyright (2019), Elsevier. (g) Image of the Nafion®/polyacrylic gel–Cu^2+^/cuprous oxide nanoparticles modified sensor equipped with a microcontroller connected to the smartphone. Adapted with permission.^47^ Copyright (2020), American Chemical Society. (h) Principle of electrochemical detection in the absence and presence of the analyte on the SPE device. Adapted with permission.^101^ Copyright (2020), American Chemical Society. Distributed under a Creative Commons Attribution License 4.0 (CC BY). (i) Fabrication of the electrochemical sensing chip under POC settings. Adapted with permission.^102^ Copyright (2021), American Chemical Society.

A few creatinine sensors seen in this review were applied to devices for point-of-care purposes. Huang *et al.* developed a complex microfluidic device integrated with mixing chambers, microvalves, and detection chambers.^31^ The samples go to the mixing chambers, where they receive pretreatment with proteinase K buffer to proteolyze the proteins. The samples are then transported to detection chambers composed of a three-electrode system, where the working electrodes were modified to detect creatinine and urea (Fig. 12a). This device was manufactured using photolithography and lift-off techniques. However, robust equipment consisting of an air compressor was required to control the valves.

Wei *et al.* used an array of 16 bare gold electrode chips (GeneFluidics, USA), where they modified the working electrodes with polypyrrole.^99^ In the device, a competitive reaction between the creatinine in the sample and an HRP–creatinine antibody is measured, observing a decrease in the signal (Fig. 12b). Liu *et al.* fabricated a microfluidic device consisting of 4 layers.^77^ The first layer made of PMMA is connected to a multi-channel peristaltic pump for blood flow, which passes to the second layer made of a dialysis membrane that only allows molecules such as creatinine, urea, and uric acid to pass, producing a solution isotonic that flows through the third layer made of PMMA. The last layer is where the sample reaches the detection chamber, where the SPEs composed of working electrodes for detecting creatinine and urea (Fig. 12c). Paper has recently emerged for application in microfluidics and paper-based analytical devices due to its porosity, small size, and portability.^100^ Cincotto and Fava used the same base in fabrication methodology.^53,65^ Two electrodes (counter/reference electrodes or working/reference electrodes) were deposited on a polyester sheet. An electrode (working/counter electrode) was deposited on another polyester sheet using the screen-printed method. Reference electrodes were modified with an Ag/AgCl ink. A filter paper cut to the desired measurements was used as a microfluidic channel, which was placed in the middle of the two modified polyester sheets to form the device (Fig. 12d and e).

Boobphahom *et al.* developed a paper-based analytical device (PAD), made on Whatman No.1 filter paper, where the base pattern of the device was printed using the wax printing method.^37^ Then the working electrode and counter electrode were screen-printed with carbon ink, and an Ag/AgCl ink was used for the reference electrode. Finally, the working electrode was modified by electrodepositing rGO, and ink with a CuO/IL composite was deposited by drop casting or an HPD300 digital dispenser (Fig. 12f). Kalasin *et al.* fabricated an SPE in which the working electrode was modified with a mixture of Nafion®/polyacrylic gel–Cu^2+^/cuprous oxide nanoparticles by drop casting.^47^ The modified SPE was connected to electronic circuit components to detect creatinine and transmit the results remotely to a smartphone (Fig. 12g). Dasgupta *et al.* used an SPE composed of a carbon working and counter electrode and an Ag/AgCl reference electrode.^101^ The compound analyte creatinine, 1-methylhydantoin, human serum albumin, and creatinine deiminase were evaluated by the principle of the enzymatic reaction of creatinine with creatinine deiminase, producing 1-methylhydantoin, which forms complexes with cobalt, increasing the current with increasing of creatinine concentration (Fig. 12h). Singh *et al.* developed a point-of-care device for detecting creatinine.^102^ First, an electrode was made with 3D printing of conductive Ag ink on a flame retardant grade 1 (FR1) PCB substrate and using a Voltera V-One conductive ink printer. One electrode was then modified with electrodeposited rGO and then with electrodeposited Cu and Fe to form the sensor. The Ag counter electrode was printed the same way as the working one. A plasma separation membrane was placed over the electrodes for the analyte to flow through (Fig. 12i).

**Fig. 13:**
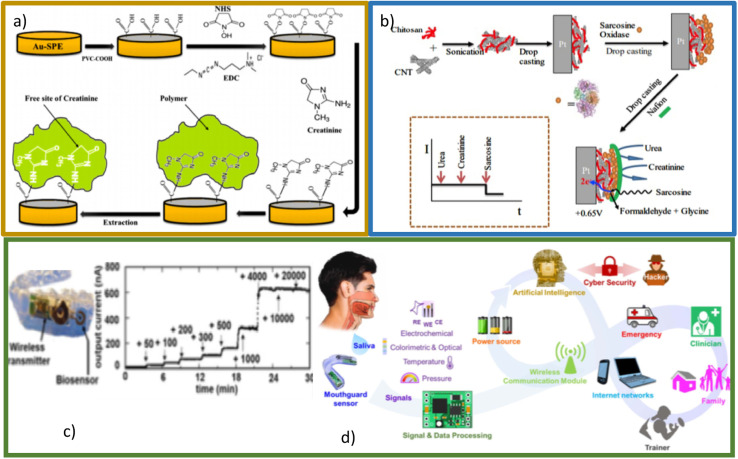
(a) Schematic representation of Au–SPE/MIP procedure. Reproduced from ref. 44 with permission from Elsevier, Copyright (2017). (b) Schematic representation of the LbL sarcosine electrochemical biosensor and idealized current time response toward analyte and interferents. Reproduced from ref. 105 with permission from Elsevier, Copyright (2018). (c) Photograph of mouthguard device (left), the response of biomarker in sample saliva (right). Reproduced from ref. 106 with permission from American Chemical Society, Copyright (2020). (d) Conceptual diagram showing important building blocks of the next generation of smart health systems. Reproduced from ref. 107 with permission from Electrochemical Society, Copyright (2022), this is an open access article distributed under the terms of the Creative Commons Attribution 4.0 License.

Molecular imprinting (MIP) is a recent technique based on the mechanism used by enzymes called the “lock and key” model.^103^ In the MIP method has higher sensitivity, stability and selective property. Furthermore, due to its low cost and relative simplicity, this method has been used more recently.^104^ A MIP-based biosensor was developed for the detection of creatinine in human urine using screen-printed gold electrodes (Au–SPE), a layer of polyvinyl carboxylic chloride (PVC–COOH) was deposited on the surface of Au–SPE, afterward, the polymerization of acrylamide and *N*,*N*-methylene bisacrylamide filled the void around it. The patterns remove binding sites within the polymer that can selectively detect creatinine at different concentrations. The molecular identification was quantified using voltammetry, electrochemical impedance spectroscopy, and spectrophotometry. Simplicity of operation, highly selective recognition ability, low cost, and small size are notable in this platform (Fig. 13a).^44^ Functional layer-by-layer (LbL) electrochemical system has shown effective detection of creatinine^105^ (Fig. 13b). Han *et al.* have developed a modified electrode based on phosphotungstic acid using the LbL method, using an electrode to determine creatinine directly with the help of copper(ii) by measuring the peak redox current of the Cu(ii)–creatinine complex/Cu(i)–creatinine complex getting good results.

One of the most interesting developments in the sensing of biomarkers (among creatinine is included) is the so-called lab-in-a-mouth^106,107^ (Fig. 13c and d). This technology would allow permanent monitoring in real time of the levels of biomarkers of interest present in saliva, which, connected to the internet of all things (IoT) would allow an early diagnosis. For biomarkers such as glucose, these devices already have an important development, however it is a perspective that is attracting much attention for portable and wearable creatinine detection devices.

### 5.1. Perspectives of modern POC devices

Even when great advances have been made in the development of sensors and their integration within POCs, however, these devices undoubtedly need further improvement and much more intensive evaluations towards a true POC applications in health care. Clinical decision making involves creatinine levels are currently based on the Jaffé method and, to a lesser extent, in other commercial devices based on the amperometry monitoring of creatinine enzymatic reaction products. Consequently, the analysis of creatinine has not yet been centralized in clinical laboratories, which blocks the acquisition of information in real time and therefore delaying the associated medical action.^108^ Evident key facts highlighted here may contribute to the establishment of new routes towards a definitive solution for the creatinine discernment in sanitary applications. Experts in the field have pointed out the drawbacks associated with using Jaffé's method and because of that, new detection principles have been actively proposed. Furthermore, it is important to note that other biological fluids with great potential for creatinine detection have not yet been fully explored. In recent times there are new strategies to achieve such as oral or based on the fingerprint technique. However, there are a number of weaknesses in the way that sensors are characterized, mainly related to calibration, potential for mass production and suitability for identification and quantification, determination of interferences from and harmful concentrations, which need to be addressed in the near future when developing new devices. Point-of-care devices have become increasingly present in the electrochemical detection of creatinine, using different methodologies with which their manufacture is more feasible. However, more research is still needed for them to become mass-produced and usable by patients, medical personnel, and laboratories.

## 6. Conclusions

Advances in the investigation of new materials for the electrochemical detection of creatinine are promising. The relevance of nanomaterials has been increasingly marked due to their use in enzymatic sensors, especially in non-enzymatic sensors. The intrinsic properties of nanomaterials, such as size and shape, and the use of support nanomaterials help to have a higher surface area, a higher surface area/volume ratio, and an increase in surface energy, which leads to a high charge transfer and catalytic activity. This has helped to improve the sensitivity, reproducibility, and lower detection limits of electrochemical creatinine sensors. However, other aspects of nanomaterials have not been exploited, such as crystallographic planes, bimetallic materials, and even exploring more forms of nanomaterials. Future research can focus on taking advantage of these aspects of the materials to improve the characteristics of the sensors and understand the interactions between creatinine and the material at the nanoscale.

## Conflicts of interest

There are no conflicts to declare.

## Supplementary Material
