# Correction: Electrochemical creatinine detection for advanced point-of-care sensing devices: a review

**DOI:** 10.1039/d2ra90109a

**Published:** 2022-11-07

**Authors:** Carlos Luis Gonzalez-Gallardo, Noé Arjona, Lorena Álvarez-Contreras, Minerva Guerra-Balcázar

**Affiliations:** Facultad de Ingeniería, División de Investigación y Posgrado, Universidad Autónoma de Querétaro Querétaro C.P. 76010 Mexico minbalca@yahoo.com.mx; Centro de Investigación y Desarrollo Tecnológico en Electroquímica S.C. Sanfandila, Pedro Escobedo Querétaro C.P. 76703 Mexico; Centro de Investigación en Materiales Avanzados S.C. Complejo Industrial Chihuahua Chihuahua C.P. 31136 Mexico lorena.alvarez@cimav.edu.mx

## Abstract

Correction for ‘Electrochemical creatinine detection for advanced point-of-care sensing devices: a review’ by Carlos Luis Gonzalez-Gallardo *et al.*, *RSC Adv.*, 2022, **12**, 30785–30802, https://doi.org/10.1039/D2RA04479J.

The authors regret the omission of a funding acknowledgement in the original article. This acknowledgement is given below.

The authors would like to acknowledge the financial support from Centro de Investigación en Materiales Avanzados S.C, Chihuahua, Mexico with the project number PI-22-05/2022.

The Royal Society of Chemistry apologises for these errors and any consequent inconvenience to authors and readers.

## Supplementary Material

